# Lu-177-PSMA dosimetry for kidneys and tumors based on SPECT images at two imaging time points

**DOI:** 10.3389/fmed.2023.1246881

**Published:** 2023-11-13

**Authors:** Gefei Chen, Zhonglin Lu, Han Jiang, Ali Afshar-Oromieh, Axel Rominger, Kuangyu Shi, Greta S. P. Mok

**Affiliations:** ^1^Biomedical Imaging Laboratory (BIG), Department of Electrical and Computer Engineering, Faculty of Science and Technology, University of Macau, Taipa, Macau SAR, China; ^2^PET-CT Center, Fujian Medical University Union Hospital, Fuzhou, Fujian, China; ^3^Department of Nuclear Medicine, Bern University Hospital, University of Bern, Bern, Switzerland; ^4^Ministry of Education Frontiers Science Center for Precision Oncology, University of Macau, Taipa, Macau SAR, China

**Keywords:** SPECT, Lu-177 PSMA, curve fitting, single time point, dosimetry

## Abstract

**Background:**

Personalized dosimetry for Lu-177-PSMA treatment requires multiple-time-point SPECT/CT scans to calculate time-integrated activity (TIA). This study evaluates two-time-point (TTP) methods for TIA calculation for kidneys and tumors.

**Methods:**

A total of 18 patients treated with 3.7-7.4 GBq Lu-177 PSMA-617 were analyzed retrospectively, including 18 sets of left and right kidneys, as well as 45 tumors. Four quantitative SPECT/CT (4TP) were acquired at 2 h, 20 h, 40 h, 60 h (*n* = 11), or 200 h (*n* = 7) after treatment, and they were fit bi-exponentially as reference. The TTP method was fitted by a mono-exponential washout function using two selected imaging time points for kidneys. For tumors, one uptake and one washout phase were modeled, assuming linear (type I) and same (type II) uptake phase between 0 h to the first time point and mono-exponential washout thereafter. Two single-time-point (STP) methods were also implemented for comparison. TIA calculated by TTP and STP methods were compared with reference to the 4TP TIA.

**Results:**

For the kidneys, the TTP methods using 20 h-60 h and 40 h-200 h had smaller mean absolute errors of 8.05 ± 6.05% and 4.95 ± 3.98%, respectively, as compared to other combinations of time points and STP methods. For tumors, the type I and type II TTP methods using 20h−60 h and 40–200 h had smaller mean absolute errors of 6.14 ± 5.19% and 12.22 ± 4.44%, and 8.31 ± 7.16% and 4.48 ± 7.10%, respectively, as compared to other TTP and STP methods.

**Conclusion:**

The TTP methods based on later imaging time demonstrated fewer errors than the STP methods in kidney and tumor TIA. Imaging at 20 h−60 h and 40 h−200 h could simplify the dosimetry procedures with fewer TIA estimation errors.

## Introduction

Radioligand therapy delivers lethal radiation to targeted cancer cells via radionuclide-labeled cell-targeting compound or ligand. Lu-177-prostate-specific-membrane-antigen (PSMA) targeting metastatic castration-resistant prostate cancer has been proven to prolong progression-free survival and overall survival of patients (1). Sequential post-treatment SPECT or planar scans are acquired to verify the absorbed dose in dose-limiting critical organs, such as kidneys and bone marrow, to avoid severe side effects before the following treatment cycles (2).

Exponential functions are frequently employed in personalized dosimetry to determine the time activity curves (TAC) by fitting sequential images, usually at 3–5 time points (3) over multiple days after Lu-177-PSMA injection (4–6). However, multiple-time-point imaging imposes burdens on clinics and patients. Simplified imaging protocols are desirable while still preserving the precision of the time-integrated activity (TIA) calculation (7–23). Hänscheid et al. (10) proposed a single-time-point (STP) approach for Y-90-DOTATOC, and Madsen et al. (12) proposed another STP method based on the population-based effective half-life for Lu-177 DOTATATE. However, large errors of TIA (>50%) for Lu-177-PSMA/DOTATATE are observed due to variations in the effective half-life among patients (19), and the optimal imaging time points are also likely to be different for different organs (24), even for the same patient. STP methods have been validated in Lu-177-PSMA data, and the recommended time point was 48 h for kidneys with possible error > 20% (19). Devasial et al. (20) developed a reduced time point method using population-based parameters based on previous Lu-177-DOTATATE cohorts to fit with individual kinetics, which was also evaluated in In-111-DOTATATE patient datasets and was further improved by the use of a model selection method (21). Nonetheless, population-based parameters may not work well for outlier patients and may not be feasible for clinics without an existing large clinical cohort. Fitting a mono-exponential model directly on two-time-point (TTP) Lu-177-DOTATATE/PSMA imaging data has been proposed, with comparable results to those obtained by multiple-time-point images in renal dosimetry (9, 15, 16, 18, 22, 23). However, there is no systematic comparison of the TTP and STP methods for kidneys and tumors, particularly for Lu-177-PSMA-617.

In this study, we aimed to investigate the best imaging time points of the TTP method for kidneys and tumors for Lu-177-PSMA-617. The reference TAC was fitted with four-time-point (4TP) imaging data using a bi-exponential function based on the best goodness of fit. Two STP methods were also implemented for comparison (10, 12).

## Materials and methods

### Patient population and image acquisition

This retrospective study included 18 anonymized patients with metastatic castration-resistant prostate cancer who were treated at Bern University Hospital between October 2019 and September 2021 under local ethics approval. The patient characteristics are summarized in Table 1.

**Table 1:** Patient demographics.

**Characteristics**	**Median [min, max]**
Age at the first therapy	73 [54, 82]
Weight (kg)	80 [56, 99]
Height (m)	1.76 [1.60, 1.87]
BMI (kg/m^2^)	25.37 [19.84, 30.47]
Prostate-specific antigen level (ug/L) before treatment	341 [16.9, 6946]

Patients underwent 4 SPECT/CT (Symbia Intevo16, Siemens Healthineers, Germany) at 2, ~20, ~40, and ~60 h (*n* = 11) or ~200 h (*n* = 7) after injection of 3.7–7.4 GBq Lu-177-PSMA-617, depending on weight, height, and tumor burden of the patient. Projections covering the head to the pelvis in three bed positions were collected with a primary energy window of 187–229 keV and two adjacent scatter windows of 150–187 keV and 229–274 keV. Projections were reconstructed using the ordered subset conjugate gradient algorithm with CT-based attenuation correction, decay correction, and triple-energy window scatter correction up to 60 updates (1 iteration with 12–60 subsets). A post-reconstruction Gaussian filter with sigma from 16.00–20.80 mm was applied. The SPECT reconstruction voxel size was 5.078 × 5.078 × 5.078 mm^3^, and the matrix size was 128 × 128 × varying length. The calibration factor for quantitative SPECT was 4.21–4.98 cps/MBq, which was determined from a Lu-177 point source with a known activity of 21.48 MBq placed next to the patient during acquisition. Corresponding low-dose CT (LDCT) data were acquired (100 kV, 27 mA), with a reconstructed voxel size of 1.27 × 1.27 × 2.00 mm^3^ and a matrix size of 512 × 512 × varying length.

**Figure 1:**
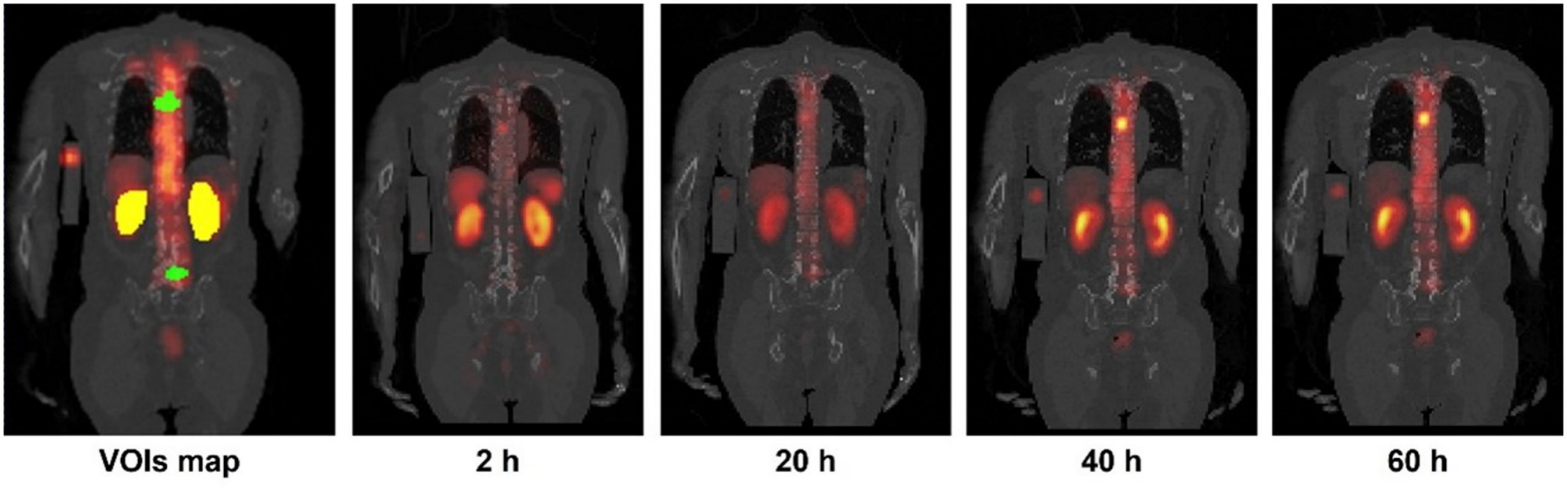
Sample kidneys (yellow) and tumor (green) maps delineated on CT and SPECT fusion images. Sample 4TP sequential SPECT/CT images acquired after Lu-177-PSMA administration are also shown.

SPECT images at the first time point (2 h) were registered and resampled to the same voxel size as the corresponding LDCT by rigid and B-spline registration with activity conservation -. Then, other time point SPECT images were registered to the first time point SPECT images using Elastix (25, 26). The left and right kidney contours were delineated slice by slice in the first time point LDCT and SPECT fusion images (Figure 1). A total of 45 tumor contours with diameter >1.5 cm and isolated from other high uptake organs were chosen by a nuclear medicine physician with 10 years of experience and then delineated based on a 40% threshold of maximum counts in the first time point SPECT images (2). The kidney and tumor maps were then applied to all registered SPECT images.

### Reference TAC

A bi-exponential function was used to fit the organ-based 4TP data for kidneys and tumors as follows:


(1) $$\begin{array}{l} \begin{array}{c} f\left(t\right)=a_{1}e^{-k_{1}t}+a_{2}e^{-k_{2}t},\; \end{array} \end{array}$$

where *a*_*i*_ is the amplitude of the exponential term and *k*_*i*_ is the effective washout or uptake rate.

**Figure 2:**
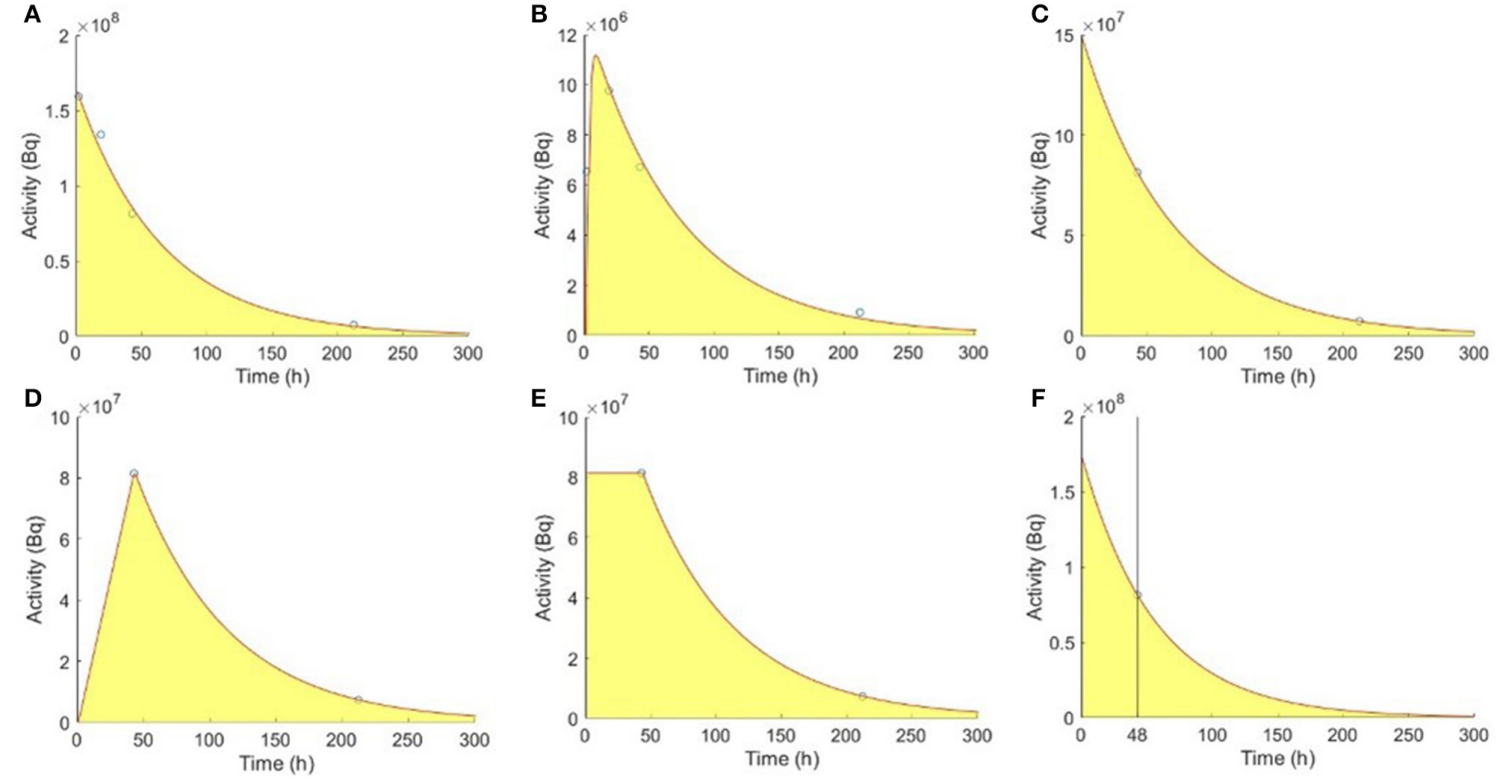
Fitting models of the reference 4TP for **(A)** kidneys and **(B)** tumors with one uptake and one washout phase. TTP methods for **(C)** kidneys, **(D)** type I tumors, and **(E)** type II tumors. **(F)** Madsen STP method for kidneys.

The TAC was fitted using a non-linear least-squares algorithm (1). Kidneys were assumed with two washout phases (Figure 2A), while tumors had one uptake and one washout phase using bi-exponential fitting of 4TP data, respectively (Figure 2B).

### TTP method

A mono-exponential function was used to fit the organ-based TTP data as follows:


(2) $$\begin{array}{l} \begin{array}{c} f\left(t\right)=ae^{-kt} \end{array} \end{array}$$

where *a* is the amplitude of the exponential term and *k* is the effective washout rate.

For kidneys, a single washout phase was modeled using the mono-exponential function to fit the SPECT data acquired at the first and second selected time points (Figure 2C). Two types of mono-exponential fitting were modeled, considering an extra uptake phase observed for tumors. Assuming no activity at 0 h, type I fitting was modeled by a linear connection between 0 h and the first time point, followed by a mono-exponential function between the first and second time points (Figure 2D). Assuming instantaneous uptake and thus that 0 h and the first time point had the same activity, type II fitting was modeled by a mono-exponential function between the first and second time points (Figure 2E) (23). The TIA for kidneys and tumors were as follows:


(3) $$\begin{array}{l} \begin{array}{c} TIA_{kidneys}=\frac{a}{k} \end{array} \end{array}$$

(4) $$\begin{array}{l} \begin{array}{c} TIA_{tumor-I}=\frac{A\left(t_{1}\right)\times t_{1}}{2}+\frac{ae^{-kt_{1}}}{k} \end{array} \end{array}$$

(5) $$\begin{array}{l} \begin{array}{c} TIA_{tumor-II}=A\left(t_{1}\right)\times t_{1}+\frac{ae^{-kt_{1}}}{k}, \end{array} \end{array}$$

where *A*(*t*_1_) is the activity at the first time point *t*_1_. All combinations of existing time points were evaluated for kidneys, i.e., 2–20 h, 2–40 h, 2–60 h, 2–200 h, 20–40 h, 20–60 h, 20–200 h, 40–60 h, and 40–200 h. For tumors, TTP pairs before 40 h were excluded to avoid the uptake phase extending to infinity, as the tumor activity concentration may peak at approximately 20 h (27).

### STP method

The TIA proposed by Madsen et al. (12) was as follows:


(6) $$\begin{array}{l} \begin{array}{c} TIA=\frac{A\left(t\right)e^{\hat{k}t}}{\hat{k}}, \end{array} \end{array}$$

where *A*(*t*) is the organ-based activity measured at imaging time *t* and $\hat{k}$ is the mean effective washout rate known from previous population-based measurements, i.e., 49.0 h and 82.0 h for kidneys and tumors, respectively (28). Accurate results can be obtained if *t* was close to or slightly larger than the patient-specific effective half-life (12). One sample using imaging time point at 48 h for kidneys is shown in Figure 2F (19).

The TIA calculated by the Hänscheid STP method (10) was as follows:


(7) $$\begin{array}{l} \begin{array}{c} TIA=A\left(t\right)\times \frac{2\times t}{\ln\;2} \end{array} \end{array}$$

If *t* fell within 0.75–2.5 times of the organ-specific effective half-life, the TIA error would be <10% (10). Only imaging time points after 30 h were considered for both STP methods in this study, as suggested in the literature (19).

### Data analysis

4TP fitting results were evaluated with the goodness-of-fit, i.e., correlation of determination R^2^. The effective half-life of the second exponential term was reported.

The percentage of mean TIA absolute error was measured for each method.


(8) $$\begin{array}{l} \begin{array}{c} Errors\;\left(\%\right)=|\frac{TIA_{\frac{STP}{TTP}}}{TIA_{4TP}}-1|\times 100\% \end{array} \end{array}$$

TTP/STP methods with mean absolute error <15% and standard deviation (STD) <10% were selected for further Bland–Altman analysis to evaluate the agreement among different fitting methods with the references for kidneys and tumors. TTP or STP methods with the narrowest 95% confidence intervals (CI) are recommended.

## Results

### Patient kinetics

The R^2^ of kidneys was 0.99 ± 0.02 (range 0.95–1.00) and that of tumors was 0.98 ± 0.02 (range 0.93–1.00). The mean effective half-life of kidneys and tumors was 49.55 ± 18.39 h (range 18.78–101.78 h) and 74.77 ± 41.12 h (range 16.95–191.24 h) for the second washout exponential terms, respectively.

### Comparison of kidney TIA

Table 2 shows TIA errors in the TTP methods compared with the reference 4TP TIA in kidneys. Combinations of 2–40 h, 2–60 h, 20–60 h, 40–60 h, and 40–200 h with a mean absolute error <15% and STD <10% were selected for further evaluation. Madsen method at 40 h and Hänscheid method at 40 h and 60 h were selected for further comparison with a mean absolute error <15% and STD <10%, as shown in Table 3.

**Table 2:** Comparison of TIA calculated by TTP methods with reference TIA for kidneys.

**|TIA Error|**	**2–20 h**	**2–40 h**	**2–60 h**	**2–200 h**	**20–40 h**	**20–60 h**	**20–200 h**	**40–60 h**	**40–200 h**
Mean ± std (%)	29.34 ± 21.49	11.83 ± 9.71	10.20 ± 8.15	24.84 ± 32.34	17.32 ± 27.61	8.05 ± 6.05	8.71 ± 14.98	8.72 ± 5.37	4.95 ± 3.98
[min, max] (%)	[0.29, 98.77]	[0.08, 40.02]	[0.21, 25.35]	[3.71, 132.21]	[0.04,125.72]	[0.56,18.14]	[0.07,57.77]	[0.73,18.13]	[1.25,14.30]
>10%	28 (77.78%)	17 (47.22%)	9 (40.91%)	11 (78.57%)	17 (47.22%)	7 (31.82%)	3 (21.43%)	8 (36.36%)	2 (14.29%)
>20%	22 (61.11%)	5 (13.89%)	4 (18.18%)	5 (35.71%)	7 (19.44%)	0 (0.00%)	1 (7.14%)	0 (0.00%)	0 (0.00%)

**Table 3:** Comparison of TIA calculated by STP methods with reference TIA for kidneys.

**| TIA error |**	**Madsen at 40 h**	**Madsen at 60 h**	**Madsen at 200 h**	**Hänscheid at 40 h**	**Hänscheid at 60 h**	**Hänscheid at 200 h**
Mean ± std (%)	9.87 ± 9.23	15.09 ± 8.91	14.81 ± 12.69	10.81 ± 9.70	10.18 ± 8.71	56.30 ± 8.50
[min, max] (%)	[0.45, 35.46]	[3.92, 33.94]	[1.21, 46.76]	[0.15,36.33]	[0.46,30.36]	[38.40,71.30]
>10%	13 (36.11%)	13 (59.09%)	10 (71.43%)	15 (41.67%)	12 (54.55%)	14 (100.00%)
>20%	5 (13.89%)	6 (27.27%)	3 (21.43%)	5 (13.89%)	3 (13.64%)	14 (100.00%)

**Figure 3:**
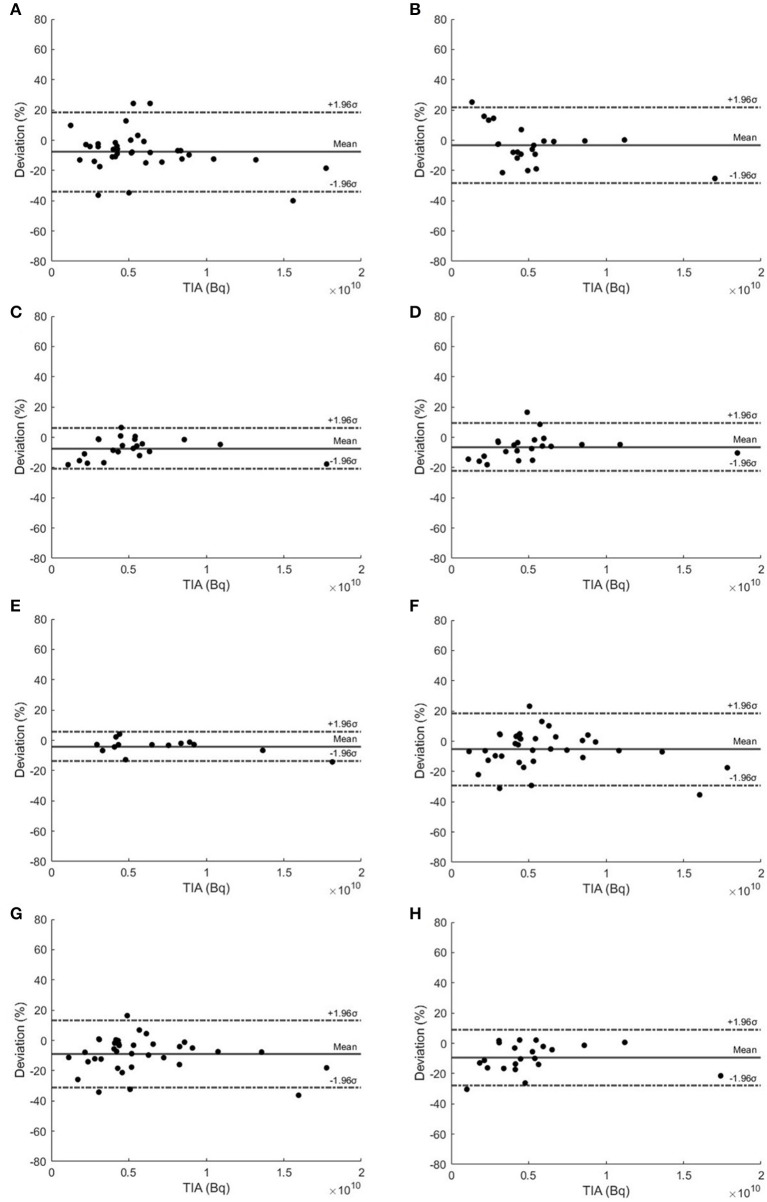
Bland–Altman plots of TTP methods using **(A)** 2–40 h, **(B)** 2–60 h, **(C)** 20–60 h, **(D)** 40–60 h, and **(E)** 40–200 h. **(F)** Madsen STP method at 40 h and Hänscheid STP method at **(G)** 40 h and **(H)** 60 h for kidneys.

The Bland–Altman plots of selected TTPs and selected STPs are depicted in Figure 3. TTP using 20–60 h and 40–200 h were recommended due to their narrowest 95% CIs, ranging from −20.90 to 6.25% and −13.76 to 5.66%, respectively. The Madsen method at 40 h had a 95% CI ranging from −29.21 to 18.60%., the Hänscheid method at 40 h had a 95% CI ranging from −31.11 to 12.99%, and at 60 h had a 95% CI ranging from −28.04 to 8.99%.

### Comparison of tumor TIA

Two types of TTP methods for tumors compared with the reference 4TP TIA are shown in Tables 4, 5, respectively. In type I TTP, 20–60 h and 40–200 h had a mean absolute error <15% and STD <10%. In type II TTP, 20–60 h and 40–200 h also had a mean absolute error <15% and STD <10%. All STPs had a mean absolute error >10%, as shown in Table 6; therefore, they were not selected for further analysis.

**Table 4:** Comparison of TIA calculated by type I TTP methods with reference TIA for tumors.

**| TIA error |**	**2–60 h**	**2–200 h**	**20–60 h**	**20–200 h**	**40–60 h**	**40–200 h**
Mean±std (%)	64.84 ± 153.73	11.68 ± 12.16	6.14 ± 5.19	8.35 ± 16.73	23.25 ± 26.96	12.22 ± 4.44
[min, max] (%)	[1.70, 683.23]	[0.04, 44.85]	[0.07, 19.76]	[0.04, 55.67]	[1.69, 148.31]	[2.84,19.38]
>10%	16 (55.17 %)	5 (31.25%)	4 (13.79%)	2 (12.50%)	21 (72.41%)	11 (68.75%)
>20%	7 (24.14%)	3 (18.75 %)	0 (0.00%)	2 (12.50%)	12 (41.38%)	0 (0.00%)

**Table 5:** Comparison of TIA calculated by type II TTP methods with reference TIA for tumors.

**| TIA error |**	**2–60 h**	**2– 200 h**	**20–60 h**	**20–200 h**	**40–60 h**	**40–200 h**
Mean±std (%)	65.81 ± 153.59	12.45 ± 12.64	8.31 ± 7.16	16.89 ± 19.99	21.96 ± 29.68	4.48 ± 7.10
[min, max] (%)	[2.43,683.65]	[0.25,46.63]	[0.26,28.68]	[0.15,72.24]	[0.96,161.09]	[0.37,26.55]
>10%	17 (58.62%)	6 (37.50%)	11 (37.93%)	8 (50.00%)	20 (68.97%)	2 (12.50%)
>20%	9 (31.03%)	5 (31.25%)	5 (17.24%)	2 (12.50%)	16 (55.17%)	1 (6.25%)

**Table 6:** Comparison of TIA calculated by the STP methods with reference TIA for tumors.

**| TIA error |**	**Madsen at 40 h**	**Madsen at 60 h**	**Madsen at 200 h**	**Hänscheid at 40 h**	**Hänscheid at 60 h**	**Hänscheid at 200 h**
Mean ± std (%)	23.25 ± 16.62	21.83 ± 13.55	13.09 ± 6.35	24.47 ± 18.91	18.77 ± 14.80	14.69 ± 11.85
[min, max] (%)	[0.63, 66.07]	[1.73, 59.26]	[2.38, 22.49]	[0.27, 75.29]	[0.89, 62.28]	[0.44, 34.51]
>10%	34 (75.56%)	21 (79.31%)	10 (62.50%)	34 (75.56%)	21 (72.41%)	9 (56.25%)
>20%	22 (48.89%)	14 (55.17%)	2 (12.50%)	23 (51.11%)	9 (31.03%)	7 (43.75%)

**Figure 4:**
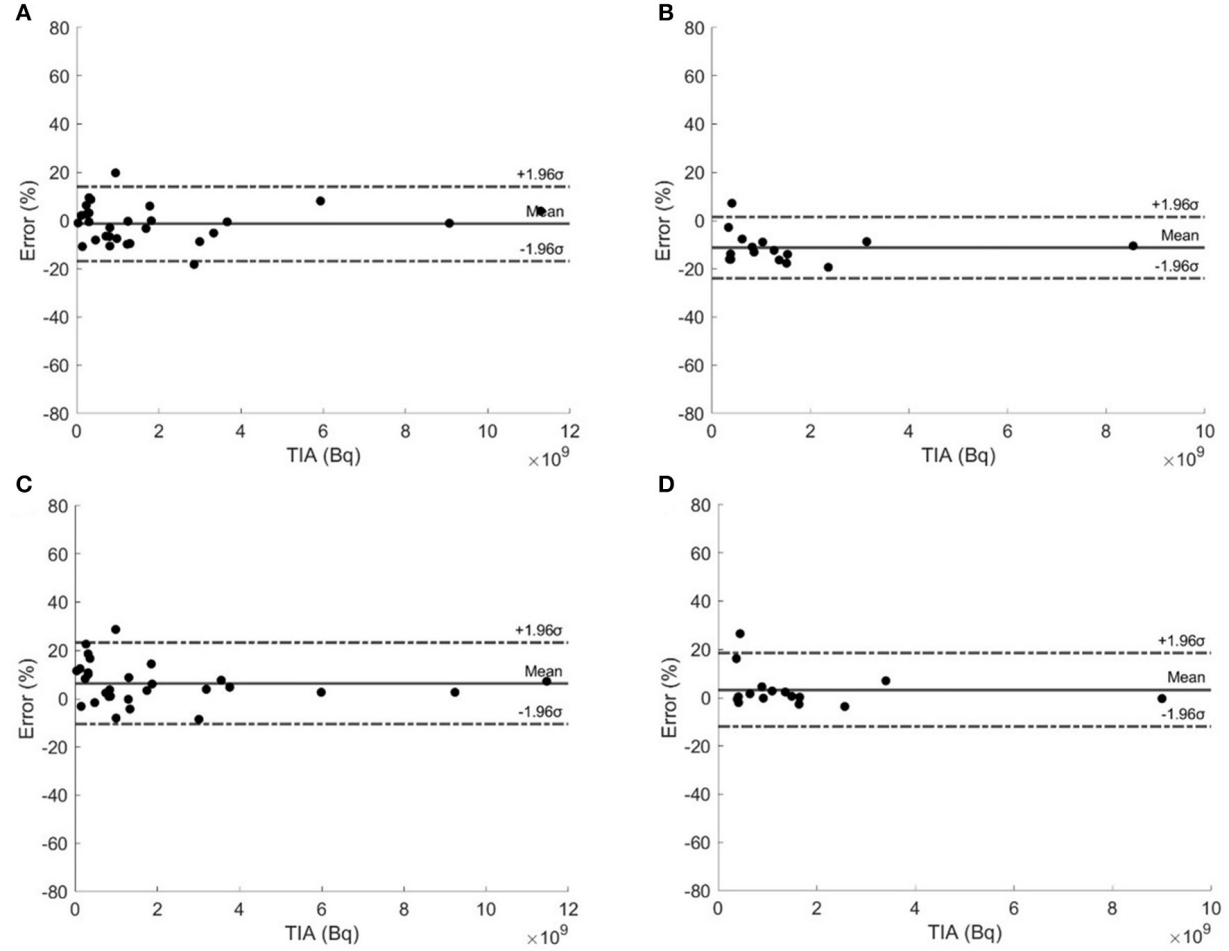
Bland–Altman plots of type I TTP methods using **(A)** 20–60 h and **(B)** 40–200 h, and type II TTP methods using **(C)** 20–60 h and **(D)** 40–200 h.

Figure 4 shows the Bland–Altman plots of selected type I and type II TTP methods. Type I TTP using 20–60 h had the narrowest 95% CI ranging from −17.08 to 14.60% and from −24.06 to 1.42%, respectively. Type II TTP methods using 20–60 h and 40–200 h had the narrowest 95% CIs, ranging from −10.12 to 22.93% and from −11.93 to 18.52%, respectively. Type I and II TTPs using 20–60 h and 40–200 h were recommended with the narrowest 95% CIs.

## Discussion

In this study, we compared the STP and TTP methods on kidneys and tumors with the reference 4TP TIA for Lu-177-PSMA-617. The STP methods could achieve better performance at 40 h, consistent with the results presented by Hou et al. (19), and better performance for tumors at >72 h, consistent with the conclusion presented by Jackson et al. (17). The accuracy of the STP methods can be improved by adding another suitable time point, i.e., TTP curve fitting, which was also reported by Peters et al. (23). The superior combination of time points was determined for kidneys and tumors based on a mono-exponential curve fitting in this study. TTP methods with 20–60 h and 40–200 h achieved better performance in kidneys and tumors, corresponding to two groups of patients with different last sampling time points. These two combinations are also implied in other studies based on Lu-177-PSMA-617 (22, 23). The proposed TTP methods could achieve an absolute 95% CI within 25% for kidneys and tumors, leading to a simplified dosimetry protocol.

Our bi-exponential model was effective for fitting all the referenced 4TP data with R^2^ > 0.93. The resultant mean effective half-life for the slow washout phase of kidneys and tumors was 49.55 and 74.77 h, respectively, consistent with existing research (28). The time integral of the slow washout phase also accounts for a large proportion of the whole TIA (10) and should be better modeled by data at later time points. Therefore, TTP methods with combinations of later time points, i.e., 20–60 h or 40–200 h, were expected to provide superior performance. They provided a mean absolute error <8% and STD <7% as well as absolute 95% CIs <25% and are recommended in the clinical protocol. Similar time combinations were implied by Rinscheid et al. (18). Our results of the TTP method in kidneys indicated a slightly worse performance than other studies (16, 18, 22), which could be attributed to different patient cohorts.

For tumors, the type I TTP method had better performance at 20–60 h, while the type II TTP method achieved better performance at 40–200 h. Our results showed better performance, with mean error <6% and STD <8%, than Resch et al. with 10 ± 14% (22). Peters et al. (23) only adopted the type II TTP method for tumors and achieved better performance with the last second time point at 168 h. The type I TTP method achieved better performance when the first time point was at 20 h, as it modeled the uptake phase better because the peak was expected to be approximately at 20 h. Therefore, the type II TTP method could overestimate the TIA for the uptake phase when the first time point was at 20 h. Therefore, in our study, the type II TTP method acquired better performance at 40–200 h.

The STP methods could achieve a mean error <10% when the selected imaging time point was within the optimal range derived from organ-specific effective half-life as proposed by Hänscheid et al. (10) and Madsen et al. (12). However, due to the high variability of the effective half-life in different patients and organs, the optimal range could vary for each patient. Moreover, the first washout phase of kidneys and the uptake phase of tumors were not modeled in the STP methods (10).

TTPs with 20–60 h and 40–200 h achieved superior performance in kidneys and tumors in this study. The 20–60 h combination could be a better choice considering the inpatient period for Lu-177-PSMA-617, as it may eliminate the need for patients to return for a second visit for the scans, allowing for a simplified dosimetry protocol. However, this study serves as a feasibility study, which was limited by a small cohort of retrospective patient data from a single center. Prospective evaluations with more patient data from different centers are warranted to validate the generalizability of our findings.

## Conclusion

TTP methods using SPECT images acquired at 20–60 h and 40–200 h could simplify the current Lu-177-PSMA dosimetry procedures with errors <19% for kidneys and <20% for tumors based on this small patient cohort.

## Data availability statement

The original contributions presented in the study are included in the article/supplementary material, further inquiries can be directed to the corresponding authors.

## Ethics statement

The studies involving humans were approved by the Institutional Review Board of Bern University Hospital. The studies were conducted in accordance with the local legislation and institutional requirements. The Ethics Committee/institutional review board waived the requirement of written informed consent for participation from the participants or the participants' legal guardians/next of kin because it's a retrospective study.

## Author contributions

GC and GM are responsible for the implementation of the method, data evaluation, and paper writing. ZL is responsible for the data evaluation. HJ is responsible for image segmentation. AA-O and AR are responsible for the clinical evaluation and data support. KS and GM are responsible for supervising the whole project. All authors contributed to the article and approved the submitted version.
